# Supplementary material for: The Mediating Effects of Marital Intimacy and Work Satisfaction in the Relationship between Husbands’ Domestic Labor and Depressive Mood of Married Working Women
Source: Int J Environ Res Public Health. 2020 Jun 24;17(12):4547. doi: 10.3390/ijerph17124547 (PMC7345565; doi:10.3390/ijerph17124547)
Supplement: Supplementary file 1 [file ijerph-17-04547-s001.zip › 20200622_IJERPH_Supplementary Tables.pdf]

## Supplementary Tables

**Table S1.** Fit index of measured model and modified model

| | $\chi^2$ | *df* | *p* | RMR | RMSEA | GFI | NFI | CFI | TLI | PRATIO | PNFI | PCFI |
|---|---|---|---|---|---|---|---|---|---|---|---|---|
| Standard | – | – | >0.05 | <0.05 | <0.05 | >0.90 | >0.90 | >0.90 | >0.90 | >0.50 | >0.50 | >0.50 |
| Measured model | 1246.572 | 318 | <0.001 | 0.022 | 0.061 | 0.886 | 0.904 | 0.926 | 0.919 | 0.906 | 0.819 | 0.839 |
| Modified model | 637.080 | 307 | <0.001 | 0.019 | 0.037 | 0.943 | 0.951 | 0.974 | 0.970 | 0.875 | 0.832 | 0.852 |

Abbreviations: *df*, degree of freedom; RMR, root mean square residual; RMSEA, root mean square error of approximation; GFI, goodness of fit index; NFI, normed fit index; CFI, comparative fit index; TLI, Tucker-Lewis index; PRATIO, parsimony ratio; PNFI, parsimony normed fit index; PCFI, parsimony comparative fit index.

**Table S2.** Result of discriminant validity

| | 1 | 2 | 3 | 4 |
|---|---|---|---|---|
| 1. Husband housework | **0.517** | | | |
| 2. Work satisfaction | 0.039 | **0.768** | | |
| 3. Depressive mood | 0.000 | 0.023 | **0.800** | |
| 4. Marital intimacy | 0.098 | 0.098 | 0.021 | **0.767** |

diagonal is AVE; AVE, average variance extracted.

**Table S3.** Comparison of fit index between hypothetical model and modified model

| | $\chi^2$ | *df* | *p* | RMR | RMSEA | GFI | NFI | CFI | TLI | PRATIO | PNFI | PCFI |
|---|---|---|---|---|---|---|---|---|---|---|---|---|
| Standard | – | – | >0.05 | <0.05 | <0.05 | >0.90 | >0.90 | >0.90 | >0.90 | >0.50 | >0.50 | >0.50 |
| Hypothetical model | 637.080 | 307 | <0.001 | 0.019 | 0.037 | 0.943 | 0.951 | 0.974 | 0.970 | 0.875 | 0.832 | 0.852 |
| Modified model | 638.324 | 308 | <0.001 | 0.019 | 0.037 | 0.942 | 0.951 | 0.974 | 0.970 | 0.877 | 0.834 | 0.855 |

Abbreviations: *df*, degree of freedom; RMR, root mean square residual; RMSEA, root mean square error of approximation; GFI, goodness of fit index; NFI, normed fit index; CFI, comparative fit index; TLI, Tucker-Lewis index; PRATIO, parsimony ratio; PNFI, parsimony normed fit index; PCFI, parsimony comparative fit index.

**Table S4.** Detail questionnaires according to measured variables.

| Latent variable | Measured variable | Questionnaire |
|---|---|---|
| Husbands' domestic labor | H1 | Food preparation |
| | H2 | Dishwashing |
| | H3 | Laundry |
| | H4 | Grocery shopping |
| | H5 | House cleaning |
| Work satisfaction | ws1 | Wage or Income level |
| | ws2 | Job security |
| | ws3 | Work |
| | ws4 | Work environment |
| | ws5 | Working hours |
| | ws6 | Potential for individual development |
| | ws7 | Workplace communication and Personal relationships |
| | ws8 | Benefits |
| | ws9 | Recognition of performance |
| | ws10 | Overall job satisfaction |
| Marital intimacy | mi1 | Frequency of conversation |
| | mi2 | Similarity of views |
| | mi3 | Marital sexual satisfaction |
| | mi4 | Trust |
| Depressive mood | dp1 | I was bothered by things that usually don't bother me. |
| | dp2 | I had trouble keeping my mind on what I was doing. |
| | dp3 | I felt depressed. |
| | dp4 | I felt that everything I did was an effort. |
| | dp5 | I felt hopeful about the future. |
| | dp6 | I felt fearful. |
| | dp7 | My sleep was restless. |
| | dp8 | I was happy. |
| | dp9 | I felt lonely. |
| | dp10 | I could not get "going." |
