# Supplementary figures and images for: The Mediating Effects of Marital Intimacy and Work Satisfaction in the Relationship between Husbands’ Domestic Labor and Depressive Mood of Married Working Women
Source: Int J Environ Res Public Health. 2020 Jun 24;17(12):4547. doi: 10.3390/ijerph17124547 (PMC7345565; doi:10.3390/ijerph17124547)

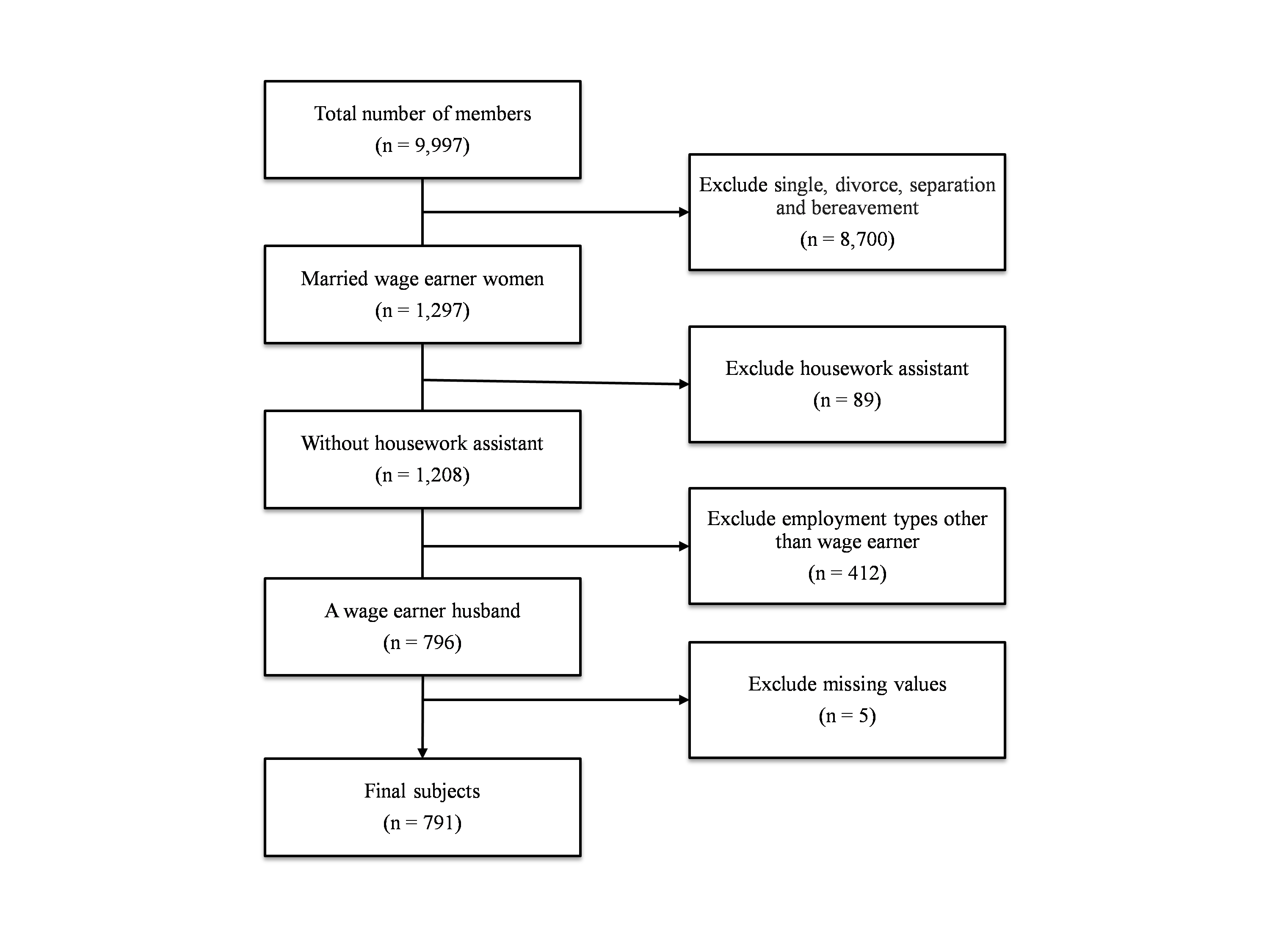

Supplement: Supplementary file 1 [file ijerph-17-04547-s001.zip › Figure S1.TIF]
